# Exploring the Fanconi Anemia Gene Expression and Regulation by MicroRNAs in Gilthead Seabream (*Sparus aurata*) at Different Gonadal Development Stages

**DOI:** 10.1007/s10126-025-10444-x

**Published:** 2025-04-11

**Authors:** Maria Papadaki, Ngoc-Son Le, Constantinos C. Mylonas, Elena Sarropoulou

**Affiliations:** 1 Biology Department, University of Crete, P.O. Box 2208, 70013 Heraklion, Crete Greece; 2 Hellenic Center for Marine Research, Institute of Marine Biology, Biotechnology and Aquaculture, Thalassocosmos, Gournes Pediados, P.O. Box 2214, 71003 Heraklion, Crete Greece

**Keywords:** Fanconi anemia genes, Teleost, Gonadal stages, miRNA, Hermaphrodite, Expression

## Abstract

Fanconi anaemia (FA) is a rare autosomal recessive disease in humans that is distributed worldwide. Fanconi anemia complementation (Fanc) proteins are essential for the appropriate functioning of the FA DNA repair pathway. They are also linked to a number of other biological processes, including oxygen metabolism, cell cycle regulation, haematopoiesis and apoptosis. So far, little research has been conducted on teleosts, but evidence shows that Fanc proteins play a significant role in immune response and sex reversal. For the examination of the expression of three *fanc* genes (*fancc, fancl,* and *fancd2*), as well as the potential regulation of these genes by microRNAs (miRNAs) in gonadal tissues at different stages of development, the present study has selected the gilthead seabream (*Sparus aurata*), a significant aquaculture species that exhibits protandrous hermaphroditism. The obtained data suggested the role of *fancl* and *fancd2* in the maturation of female gonads and the miRNAs miR-210, miR-217 and miR-10926 have been identified as putative regulators of *fancd2*, *fancc* and *fancl*, respectively. Overall, the data indicated the potential use of *fancl* and *fancd2* genes as sex biomarkers in conjunction with their respective regulation by miRNAs. To the best of our knowledge, this is the first study demonstrating the importance of *fanc* genes, along with putative regulatory miRNAs, in the reproduction of an important marine aquaculture species.

**Supplementary Information:**

The online version contains supplementary material available at 10.1007/s10126-025-10444-x.

## Introduction

Fanconi anemia (FA) is a rare autosomal recessive disease in humans, with a global prevalence. Fanconi anemia is distinguished by catastrophic bone marrow failure, which frequently manifests by five years of age (Rosenberg et al. 2008) and is often accompanied by characteristic congenital anomalies, including slow growth, short stature, microcephaly, microphthalmia, as well as hypogonadism and infertility (Kee and D'Andrea 2010). The Fanconi anemia complementation (Fanc) proteins are known to play a critical role in the proper functioning of the FA DNA repair pathway, which is essential for repairing DNA damage, particularly interstrand cross-links (ICLs). Beyond their importance in homologous recombination (HR)-mediated DNA double-stranded breaks (DSB) repair, Fanc proteins have been functionally linked to oxygen metabolism, cell cycle regulation, hematopoiesis, and apoptosis. In humans, the FA pathway/complex is constituted of 22 known genes (Tsui and Crismani 2019). In the context of teleost fishes, it has been shown that zebrafish (*Danio rerio*), which underwent an additional round of whole genome duplication to the teleost-specific whole genome duplication (TWGD) (Dehal and Boore 2005), has a single ortholog of each human *fanc* gene. Notably, mutations in at least two of them (*fancl* and *fancd1*(*brca2*)) have been observed to result in female-to-male sex reversal (Rodriguez-Mari et al. 2010; Rodríguez-Marí et al. 2011). Furthermore, investigations have demonstrated that, as in humans, zebrafish *fanc* genes are required for genome stability and for suppressing apoptosis in tissue culture cells, in embryos treated with DNA damaging agents, and in meiotic germ cells. Zebrafish has been employed as a model for investigating the disease, but as of yet, the physiological function of *fanc* genes in reproduction-related processes in fish has received little attention.

Teleost fishes represent the largest vertebrate group, comprising over 35,000 species, and exhibit a wide range of sex determination mechanisms (Kobayashi et al. 2012), including genetic sex determination, whereby sex is determined by specific genes or chromosomes (Devlin and Nagahama 2002; Nagahama et al. 2021; Kitano et al. 2024), and environmental sex determination, whereby sex is influenced by external factors such as temperature, salinity, or social conditions (Baroiller et al. 2009; Piferrer et al. 2012; Yamamoto et al. 2019). In contrast, sex differentiation encompasses the array of genetic and physiological processes that metamorphose an undifferentiated gonad into a testis or ovary (Piferrer and Guiguen 2008). Furthermore, fish may have different reproductive strategies, with some species being gonochoristic and others hermaphroditic (Devlin and Nagahama 2002), with fertilization being internal or external (Jalabert 2005) and with oviparous, viviparous and ovoviviparous offspring production (Patzner 2008). The gilthead seabream (*Sparus aurata*)*,* an important Mediterranean Sea aquaculture fish, is a protandrous hermaphrodite species, which functions as a male for the first year of reproductive life (two years old) and then a proportion of fish reverse sex to female during the third year, and thereafter, depending on the social structure of the population (Happe and Zohar 1998). It is important to note that the proportion of males remains stable at approximately 15–20%, even after the inclusion of smaller males to the population to balance the previous year's sex ratio. This is due to the fact that older, larger males tend to reverse their sex to female (Papadaki et al. 2024a). In the non-reproductive season, adult fish gonads -of both males and females- are bisexual, containing both a central female and a peripheral male part. During the following reproductive season, in male fish the testicular part proliferates and undergoes maturation (spermatogenesis and spermiation), while still showing a female part lining the central cavity of the gonad; on the contrary, in female fish gonads contain exclusively ovarian tissue (Mylonas et al. 2011).

The sex reversal process is controlled by the brain-pituitary-gonad axis, under the influence of genetic and epigenetic mechanisms (Ortega-Recalde et al. 2020). From a molecular perspective, genes that have been associated with the sex reversal process in protandrous fish include aromatase (*cyp19a1a*) -the enzyme that converts the androgen testosterone to 17β-estradiol, the major estrogen in fish-, the expression of which is likely to be triggered by an increase in luteinizing hormone receptor (*lhcgr*) gene expression (Wong et al. 2006). The sex reversal process, is also associated with other known sex-related genes, such as *dmrt1* (doublesex and mab-3 related transcription factor 1) (Liarte et al. 2007) and *amh* (anti-Müllerian hormone) (Casas et al. 2016). In recent studies, new candidate genes have been proposed to be associated to the sex reversal process, including the *fanc* subtype l (*fancl*) gene. In the context of protogynous New Zealand spotted wrasse *Notolabrus celidotus,* a species undergoing sex reversal, *fancl* was found to be downregulated in the male stages during the late transitioning and terminal phases (Muncaster et al. 2023). This finding suggests a potential role for *fancl* in the species’ sex change process. A mutation of the same gene in zebrafish, has been found to induce sex reversal from female to male through elevated DNA damage and apoptosis of primordial germ cells (Rodriguez-Mari et al. 2010). Further studies conducted more recently, including CRISPR/Cas9 knockouts have shown complete or partial female-to-male sex reversal in the knockouts of *fanc* genes (Ramanagoudr-Bhojappa et al. 2018). More specifically, authors showed that zebrafish homozygote *fancc* mutants by 45 days post fertilization (dpf) showed definitive testicular differentiation. Hence, these findings suggest that *fanc* genes may play a role in maintaining the sexual phenotype. Moreover, the loss of function of *fanc* genes may result in sex reversal or sterility in fish, both aspects with extreme interest in aquaculture, especially of marine species (Wong and Zohar 2015; Budd et al. 2015).

Given the established involvement of *fanc* genes in reproduction and immune response, it is imperative to consider the regulatory mechanisms that underpin these processes. Numerous studies have demonstrated that environmental factors and fish welfare can influence gender and immune response capacity (Makrinos and Bowden 2016; Anderson et al. 2012; Goikoetxea et al. 2017). This observation underscores the critical need to investigate the role of epigenetic regulatory mechanisms in these contexts. These include post-translational modifications such as ubiquitination and phosphorylation, as well as intricate protein interactions. Additionally, epigenetic mechanisms, such as the regulation by microRNAs (miRNAs), may play a pivotal role in this process. MiRNAs are environmentally controlled non-coding RNAs that have been demonstrated to target, among other mRNAs, those involved in reproduction-related processes, including germ cell differentiation, gametogenesis, steroidogenesis, and apoptosis (Gay et al. 2018; Janati-Idrissi et al. 2024; Papadaki et al. 2020, 2024b; van Gelderen and Ribas 2024). The involvement of miRNAs in the sex reversal of various species has also been a subject of investigation (Liu et al. 2015), as is the regulation of *fanc* genes by miRNAs in mammals (Cappelli et al. 2024; Degan et al. 2019).

In the present study, we examined the existence of a direct link between miRNAs to *fanc* gene regulation in gilthead seabream, in relation to its reproductive tissues (ovaries and testes). Specifically, expression of *fancc*, *fancd2* and *fancl* were investigated in (a) the mature and active ovary of female fish, (b) the inactive and immature ovarian part of male fish, and (c) the active and mature testicular part of male fish. Furthermore, a functional screening approach was devised to identify differentially expressed miRNAs in the three different gonad tissues and computational analysis identified putative miRNAs that target the *fancc*, *fancl* and *fancd2* genes.

## Materials and Methods

### Ethics Approval

Ethical approval for the study was obtained by the relevant Greek authorities (National Veterinary Services) under license No 255356 (ΑΔΑ: 6Λ4Σ7ΛK-ΩΜΥ). All procedures involving animals were conducted following the “Guidelines for the treatment of animals in behavioral research and teaching” (Anonymous 1998), the Ethical Justification for the use and treatment of fishes in research: An update (Metcalfe and Craig 2011), and the “Directive 2010/63/EU of the European Parliament and the Council of 22 September 2010 on the protection of animals used for scientific purposes” (EU 2010).

### Fish Sampling

Three female and three male gilthead seabream aged 6 years old were sacrificed during the spawning season of the species (February 2019). The two different parts of the male gonad (mature and active testes, M, and immature and inactive ovary, fM) and the mature and active ovaries of females (F) were collected. One portion of the gonad was kept in 4% formaldehyde:1% glutaraldehyde (McDowell and Trump 1976) for histological processing, and another one was submerged in RNAlater (Sigma-Aldrich, Germany) and transferred to −80 °C until RNA extraction.

### Histology

For histological processing, gonads were dehydrated in a 70–95% ethanol series and embedded in glycol methacrylate resin (Technovit 7100, Heraeus Kulzer, Germany). Serial sections were obtained at 4 µm thickness on a semi-automatic microtome (Leica RM2245, Germany). Staining of the sections was performed with methylene blue/azure II/basic fuchsin (Bennett et al. 1976) and stained slides were examined under a light microscope (Nikon Eclipse 50i, Japan).

### RNA Extraction and Evaluation

Total RNA extraction of all gonad samples was carried out by disrupting 30 mg of tissue samples in liquid nitrogen with mortar and pestle, followed by sample homogenization by passing the lysate five times through a 23-gauge (0.64 mm) needle. Homogenized samples were further processed by applying the Nucleospin miRNA kit (MachereyNagel, Duren, Germany) following the manufacturer's instructions. The RNA quantity was estimated with a Nano-Drop ND-1000 spectrophotometer (NanoDrop Technologies, Wilmington, DE), and the RNA integrity was evaluated by 1.5% agarose gel electrophoresis as well as by running DNAnalyzer RNApicoChip (Agilent 2100 Bioanalyzer, Agilent Technologies, Santa Clara, CA, USA). The RNA integrity number (RIN) has been shown not to be applicable for fish gonads (Rojo-Bartolome et al. 2016, 2017b; Shen et al. 2017), due to the high accumulation of 5S RNA in these tissues (Fig. 2).

### Quantitative Real-Time PCR and Data Acquisition

Complementary DNA (cDNA) was synthesized from 1 μg of total RNA by using the PrimeScript™ RT reagent Kit with gDNA Eraser (Takara Bio, Saint-Germain-en-Laye, France). For quantitative real-time PCR (qPCR), cDNA was diluted 1:50 with RNAse-DNAse free water (Sigma-Aldrich, Germany) and stored at −80 °C. The expression levels of three selected *fanc* genes (*fancl*, *fancc* and *fancd2*) in the F, M and fM gonads were assessed by qPCR. Primers for the genes were designed applying Primer3 (http://frodo.wi.mit.edu/primer3) and Beacon Designer 8.0 software (PREMIER Biosoft International, USA). All primer pairs were evaluated for their specificity by melting curve analysis prior to quantification. The product sizes, and annealing temperatures for all primer pairs are listed in Table 1. Real-time qPCR was performed by applying the MIC qPCR cycler detection system (Bio Molecular Systems, Australia). Reactions were initiated by mixing 5 μL 1:50 of diluted cDNA of each sample with 10 μL of SYBR® FAST qPCR Master Mix (Kapa Biosystems, Woburn, MA, USA) as the fluorescent intercalating agent, 0.4 μL of specific forward and reverse primer (10 μM) and 4.2 μL H_2_O (end volume 20 μL). The thermal profile for all reactions was 3 min at 95 ^◦^C, followed by 40 cycles of 10 s at 95 °C and 30 s at 55 °C (annealing and extension step). After each cycle, a plate reading for fluorescent signal assessment was carried out to perform a dissociation curve analysis with a gradient of 50 °C to 95 °C. Negative controls (RT^−^) were routinely used for each primer set. Ct- values were obtained by setting within the MIC software the export parameters LinReg and autoTreshhold. Three candidate reference genes (*eef1a*, *L13,* and *18S*) were evaluated as reference genes and *eef1a* and *18 s* were chosen as reference genes based on NormFinder and geNorm. The relative quantity was determined by the 2^ΔΔCT^ method (Livak and Schmittgen, 2001). Obtained expression data were tested for normality and homogeneity of variances using Shapiro–Wilk and Levene's tests. In instances where the requisite criteria (normality and homogeneity) were not met, non-parametric tests were employed for analysis. These comprised a Kruskal–Wallis ANOVA on ranks, followed by a Mann–Whitney rank sum with the asymptotic significance (2-tailed) p-value set to P < 0.05. The analyses were conducted using the SPSS-PC release 17.0 software (SPSS Inc., Chicago, IL, USA). Gene expression data are presented as the mean ± standard error of the mean of the three biological replicates.

**Table 1:** Primer pair sequences, amplicon sizes (bp), annealing temperatures (Ta, ^◦^C), and Gene accession numbers for genes used for real-time PCR

Acronym	Forward primer	Reverse primer	Bp	Ta (°C)	Accession number
*18S*	AGGGTGTTGGCAGACGTTAC	CTTCTGCCTGTTGAGGAACC	164	55	AM490061.1
*EF1a*	AAATGCGGAGGAATCGACAA	GAGCCCTTGCCCATCTCAG	71	55	AF184170.1
*fancd2*	GGTGGTGTGTAGTCTTCG	ATCAATGTAACGCTGTCC	153	55	XM_030420752.1
*fancl*	CACTGAGGAATAAACTGAAC	CGAGACGATAGGAGTAAC	147	55	XM_030442506.1
*fancc*	GTCTGGTCTAGTTGATGAAG	TTCTCAGCAGGACTCTATAC	156	55	XM_030415875.1

### Small Non-Coding RNA (sncRNA) Libraries Construction and Sequencing

sncRNA libraries were generated from 1 μg total RNA using the NEBnext multiplex Small RNA Library Preparation kit for Illumina sequencing (New England Biolabs, Ipswich, MA, USA). Size fraction was carried out according to the manufacturer's recommendations by running a polyacrylamide gel (6% TBE gel, Lonza, Basel, Switzerland) at 4 °C for 1 h. Each sample was tagged with a different multiplex identifier tag provided by NEB. The generated sncRNA libraries were evaluated by DNA high-sensitivity chips (Bioanalyzer, Agilent) and quantified by Qubit (Life Technologies, Carlsbad, CA, USA) as well as by the DNA high-sensitivity chip measurements. Differentially indexed libraries were pooled at a concentration of 4 nM and single-strand sequenced over 4 lanes on the Illumina NextSeq500 sequencing platform at the Genomics Facility of the Institute of Molecular Biology & Biotechnology, Forth, Crete, Greece.

### Sequencing Reads Analysis

All reads were submitted to quality control using the open-source Fastqc v0.10.0 software (http://www.bioinformatics.babraham.ac.uk/projects/fastqc). Sequencing reads were quality and adapter trimmed applying Trimmomatic software 0.30 (Bolger et al., 2014) and imported into the CLC genomics Workbench (v10.1). Putative sncRNAs were further extracted, and all reads were counted accordingly. The minimum sampling count (the number of copies of the raw sncRNAs reads included in the resulting count table) was set to 5. The annotation of putative miRNAs was achieved through the alignment of obtained sequencing reads with the teleost miRNAs listed in miRBase (release 21.1). with the following order, A*statotilapia burtoni, Oryzias latipes, Tetraodon nigroviridis, Fugu rubripes, Danio rerio, Cyprinus carpio, Gadus morhua, Hippoglossus hippoglossus, Paralichthys olivaceus, Ictalurus punctatus, Salmo salar, Petromyzon marinus,* as well as against the mammals *Gorilla gorilla, Homo sapiens, and Mus musculus* (Griffiths-Jones et al. 2008) and against the three-spined stickleback (*Gasterosteus_aculeatu*s) sncRNA database Gasterosteus_aculeatus.BROADS1.ncrna. Subsequently, the merging of variants of the same miRNAs was performed, which resulted in a list of “sampled grouped” transcripts with the corresponding read count. 

### MiRNA Differential Expression Analysis

Differential expression (DE) analysis of miRNAs was assessed by DeSeq2 implemented in SarTools version 1.2.0 (Varet et al., 2016) with default parameters. Transcripts with padj. < 0.05 and log2fold change (log2FC) >|1| were considered as differentially expressed. Principal Component Analysis (PCA), was carried out in R (R Core Team 2022). Heatmap analysis was carried out using the Heatmapper application (Babicki et al. 2016). Among the most abundant differentially expressed miRNAs, the ones that showed the opposite pattern of expression than the *fanc* genes in each of the three comparisons were further investigated.

### Identification of MiRNAs Targeting mRNAs of Differential Expressed *Fanc* Genes

Differentially expressed and characterized miRNAs from each gonadal maturation stage that putatively target *fanc* genes (*fancl*, *fancc* and *fancd2*) were identified. Therefore, the gilthead seabream 3’UTRs were retrieved from the Ensembl archive 111 (January 2024), and the hybridization dynamics were assessed by applying RNAhybrid, version 2.12 (Kruger and Rehmsmeier 2006), with the energy threshold set to mfe ≤ − 20.

## Results

### Histology

**Fig. 1:**
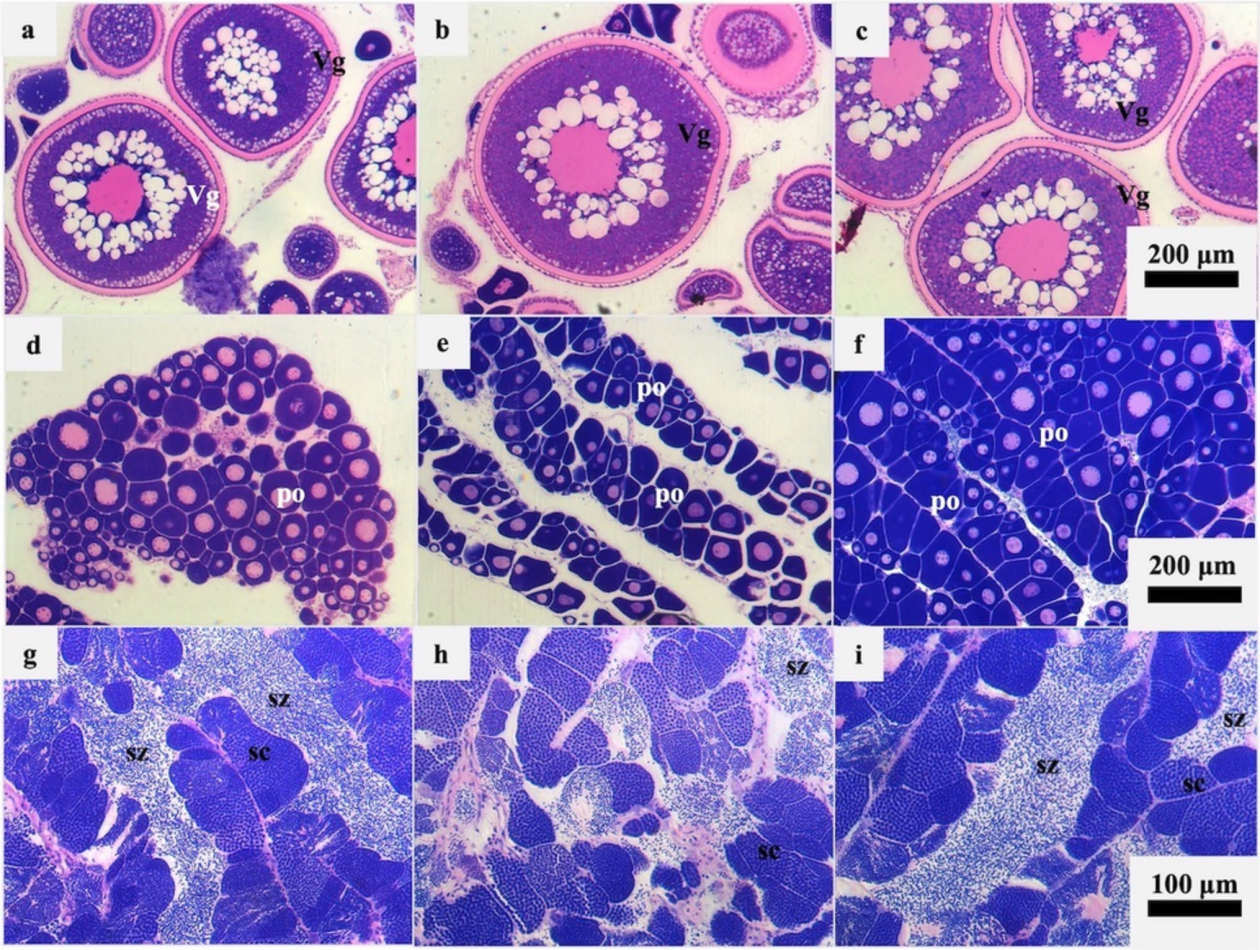
Histological sections of ovaries and testes of 6-year-old gilthead seabream during the reproductive period (February). **a**, **b** and **c**. Group F ovaries collected from mature females with vitellogenic oocytes (Vg); **d**, **e** and **f**. Group fM ovaries (inactive and immature ovarian part of male fish), consisting exclusively of primary oocytes (po); **g**, **h** and **i**. Group M testes (active and mature testicular part of male fish) containing spermatocytes (sc) and spermatozoa (sz). The scale bars of each row correspond to the three photographs of the same row (200 μm for photographs a-f and 100 μm for photographs g-i)

**Fig. 2:**
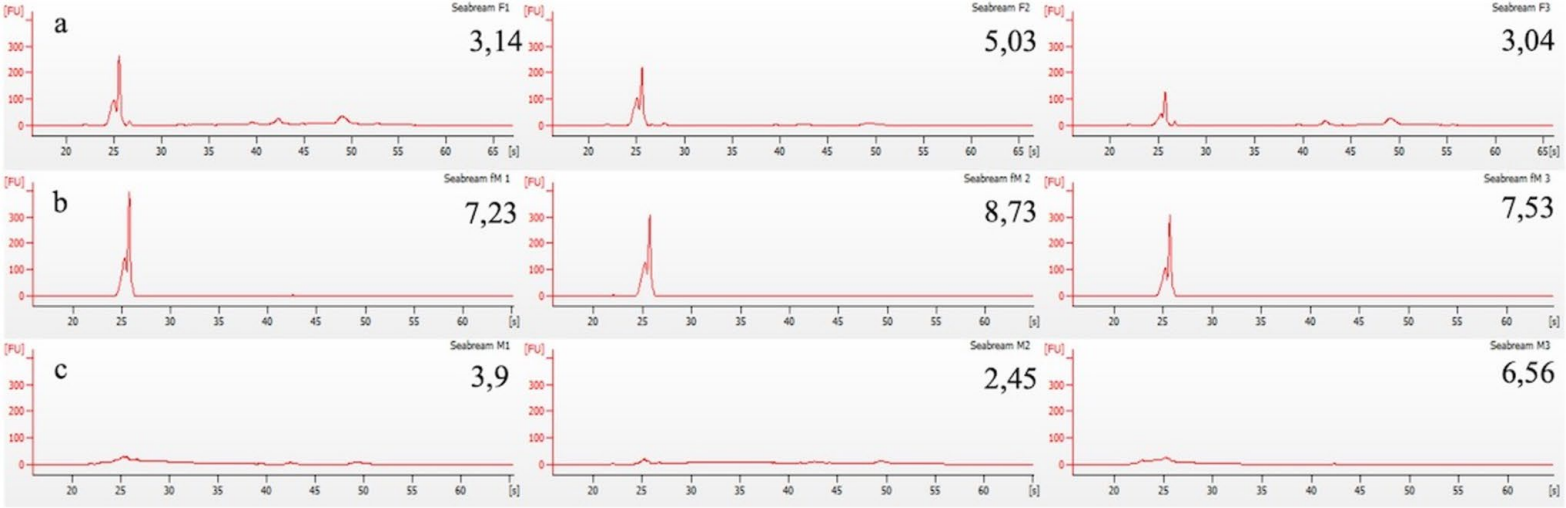
Total RNA profiles in DNAnalyzer from mature female gonads (group F, **a**), inactive and immature ovarian part of male fish (fM, **b**) and active and mature testicular part of male fish (M, **c**) of 6-year-old gilthead seabream collected during the reproductive season (February). The numbers shown at the top right of each graph represent the 5S/18S index

The ovaries of F individuals were found to contain oocytes at the vitellogenesis stage (Fig. 1a, b and c), whereas immature and inactive female parts (fM) of male gonads contained only primary oocytes (Fig. 1d, e and f). The mature and active parts of male gonads (M) contained spermatocytes at different stages of spermatogenesis, as well as free spermatozoa in the testicular lumen (Fig. 1g, h and i). Histological results agreed with the high 5S rRNA peak at approximately 180 nt (~ 25 s) of the DNAnalyzer profile. In male individuals, the 5S/18S index showed variation among the three samples (Fig. 2).

### Relative Gene Expression Analysis of *Fanc* Genes

**Fig. 3:**
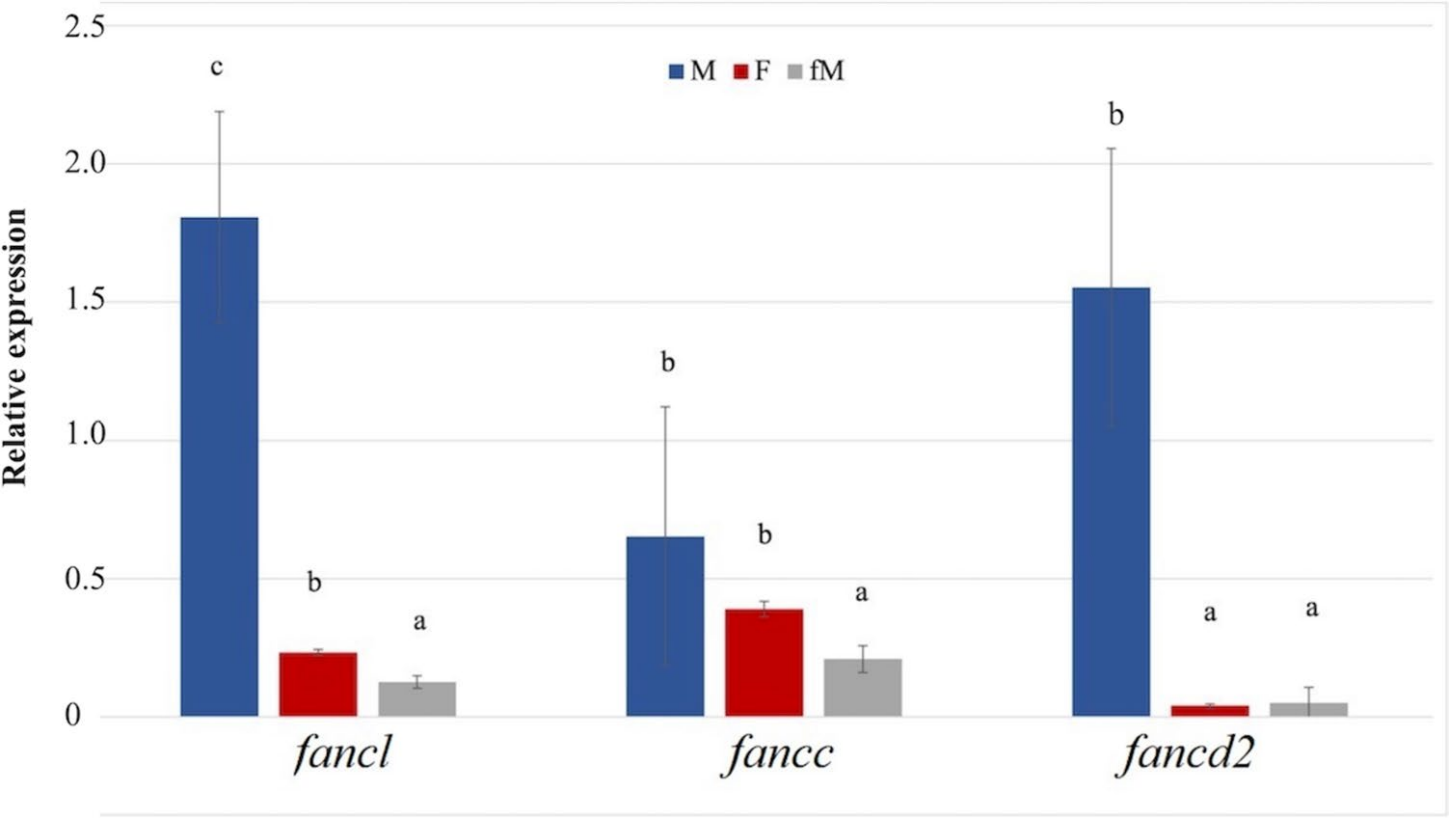
Differential expression values of *fancl*, *fancc* and *fancd2* genes among M, F and fM gonads of the gilthead seabream during the reproductive season determined by qPCR. Different letters indicate statistically significant differences in the expression of *fanc* genes among the three gonadal types (*p* < 0.05). Values are presented as mean ± SEM (n = 3)

The results of the quantitative gene expression analysis indicated that the three *fanc* genes were generally highly expressed in male gonads. In particular, the expression of *fancl* and *fancd2* was significantly higher in the male gonads than in both female gonads. In contrast, the expression of *fancc* was only significantly lower in the immature female gonads (fM) compared to the male gonads. The analysis of the male gonads revealed variability in the *fancc* expression. The comparison of the mature female gonads (F) to the fM revealed a significantly higher expression of *fancl* and *fancc* in the mature female gonads (Fig. 3).

### MicroRNAs Analysis

**Fig. 4:**
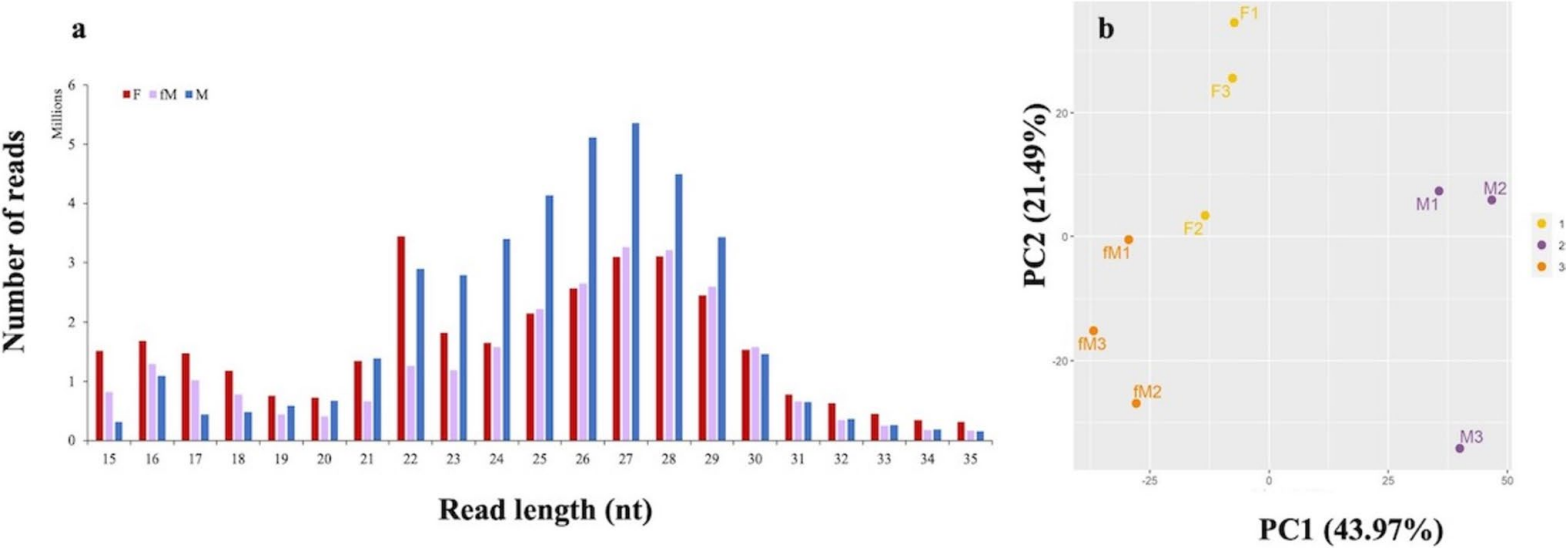
**a** Read length distribution of small RNA sequences from 6-year-old gilthead seabream gonads during the reproductive season. Red: mature ovary (F), purple: inactive and immature ovarian part of male fish (fM), blue: active and mature testicular part of male fish (M). **b** PCA analysis of sncRNAs of mature female gonads (F-1, F-2, and F-3), inactive and immature ovarian part of male fish (fM-1, fM-2 and fM-3) and active and mature testicular part of male fish (M-1, M-2 and M-3) of 6-year-old seabream. The first principal component (PC1) is expected to discriminate samples from different biological conditions. The first two components of the PCA are presented, with the percentages of variance associated with each axis

In total about 117 million raw reads were obtained with an average of 13 million raw reads per sample. After trimming, around 100 million reads were received with an average of 11 million reads per sample (Suppl. Table 1). The distribution of read lengths revealed two main peaks for all gilthead seabream gonad groups, one at 21–23 nt and one at 24–30 nt (Fig. 4a), with the former corresponding to putative miRNAs and the latter most probably to putative piRNAs. With respect to the expression profiles of sncRNAs, principal component analysis (PCA) distinguished the three gonadal groups, with the F and fM stages exhibiting a closer proximity to one another compared to the M stage (Fig. 4b).

**Fig. 5:**
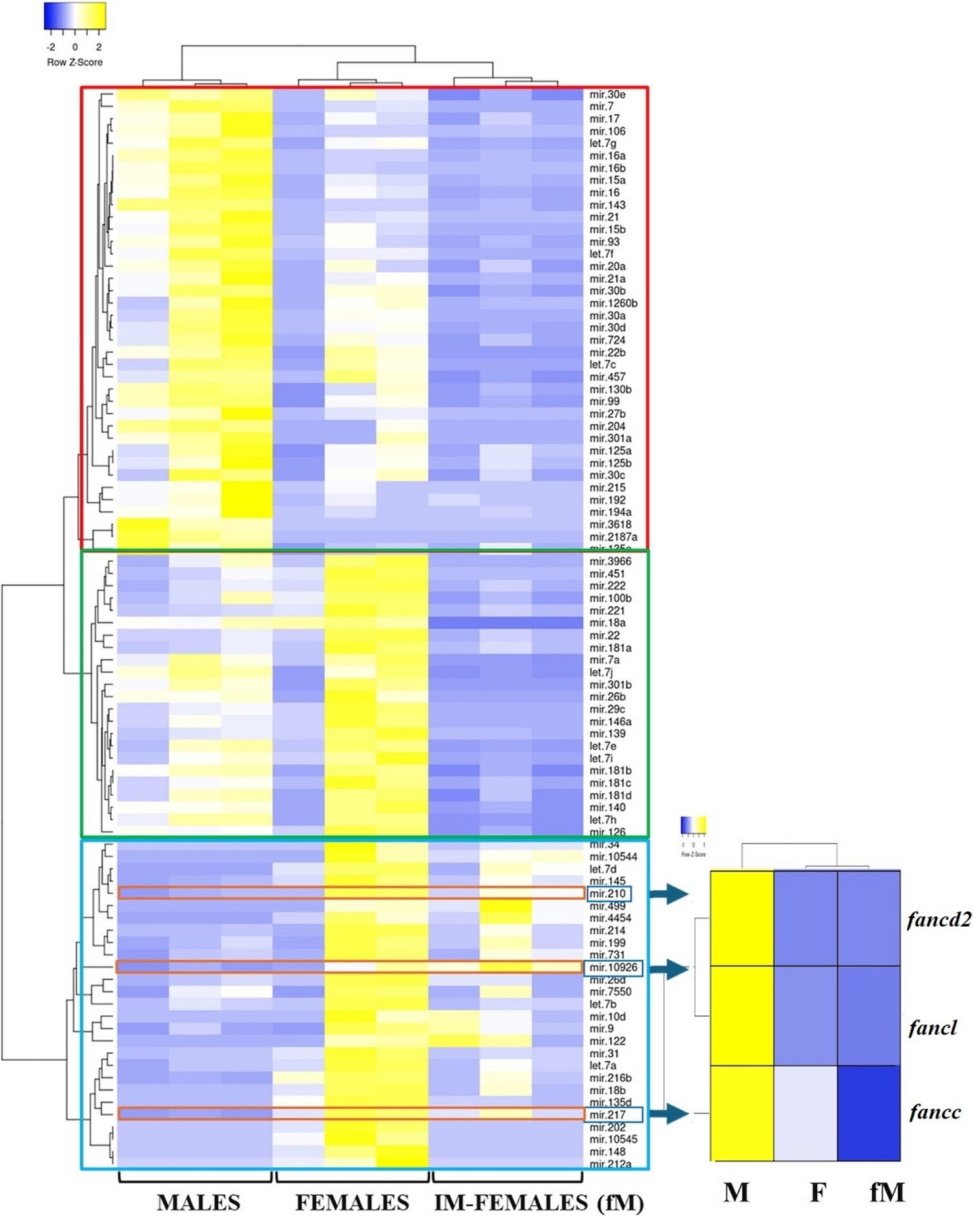
Hierarchical clustering of the differentially expressed miRNAs among mature and active male parts of the male gonads (M), the mature female gonads (F) and the immature and inactive ovarian parts of male gonads (fM) of sharpsnout seabream (padj < 0.005) collected during the reproductive season. Individual gonadal samples of each group (M F, and fM) are indicated at the bottom of each column. Each row represents the expression of one miRNA and blue and yellow represent low and high abundance, respectively. The expression of the *fanc* genes, being putatively targeted by miR-210, miR-10926 and miR-217 are shown on the right

**Fig. 6:**
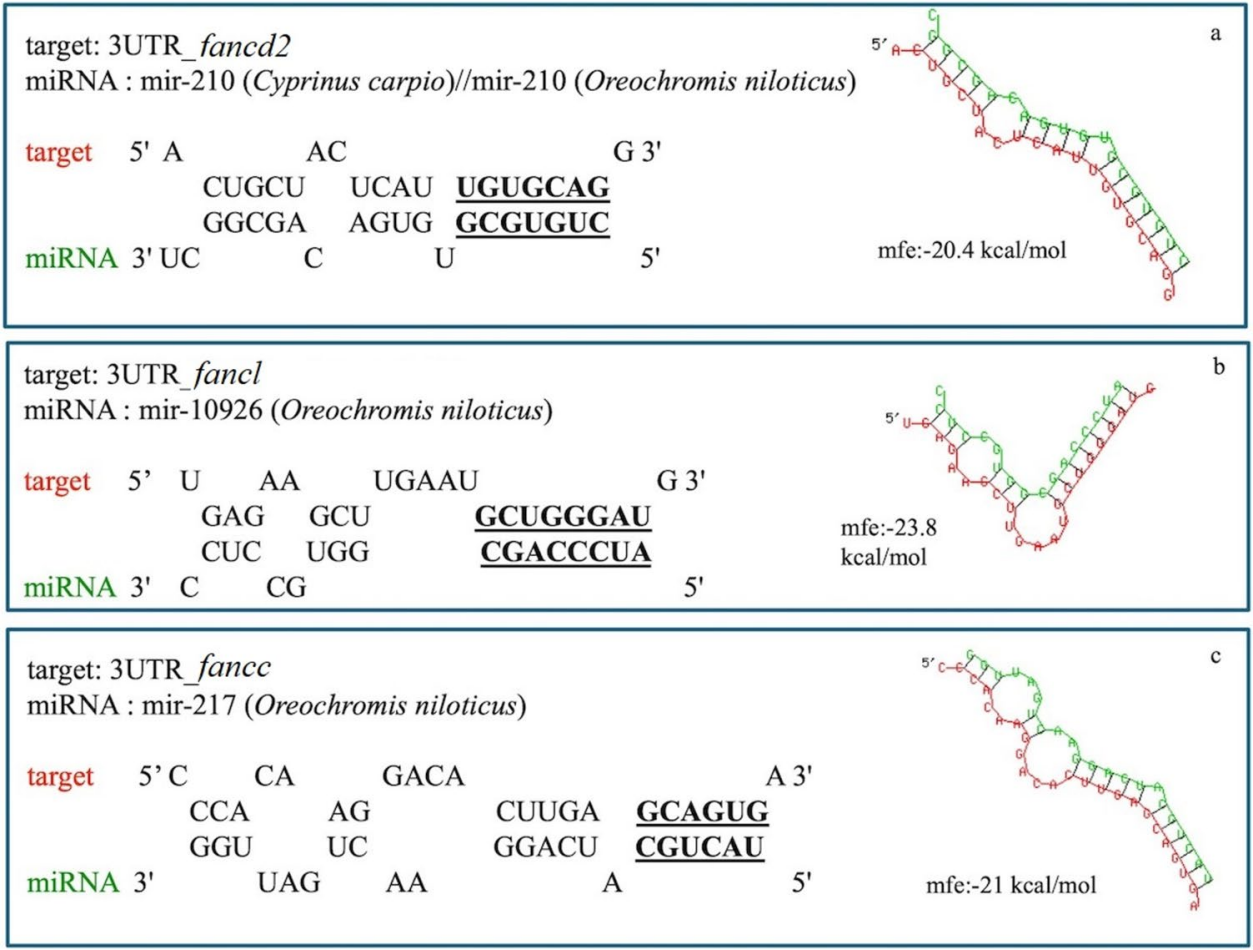
Putative hybidization of (**a**) miR-210 with the 3’ untranslated region (3’UTR) of *fancd2*, (**b**) miR-10926 with the 3’ UTR of *fancl* (**c**) miR-217 with the 3’ UTR of *fancc*, applying RNAhybrid analysis, with minimum freedom energy (mfe) of < −20 and with complete seed region complementarity

Reads identified as miRNAs with a significance threshold of DE padj. < 0.05 and log2FC >|1| (Suppl. Table 2) revealed three different clusters: a “male-biased” cluster including 38 miRNAs in higher abundance in M gonads, a “mature female-biased” cluster including 23 miRNAs in higher abundance in F gonads and a “immature female-biased” cluster with 27 miRNAs in higher abundance in fM gonads (Fig. 5). In the present study, the potential regulatory role of miRNAs in the three *fanc* genes under study was investigated. Following this investigation, three miRNAs were selected, i.e. miR-210, miR-10926, and miR-217, suggesting their potential functionality in targeting *fancd2*, *fancl*, and *fancc*, respectively (Fig. 5). Hybridization and mfe values for these three miRNAs with the respective *fanc* genes are shown in Fig. 6.

**Fig. 7:**
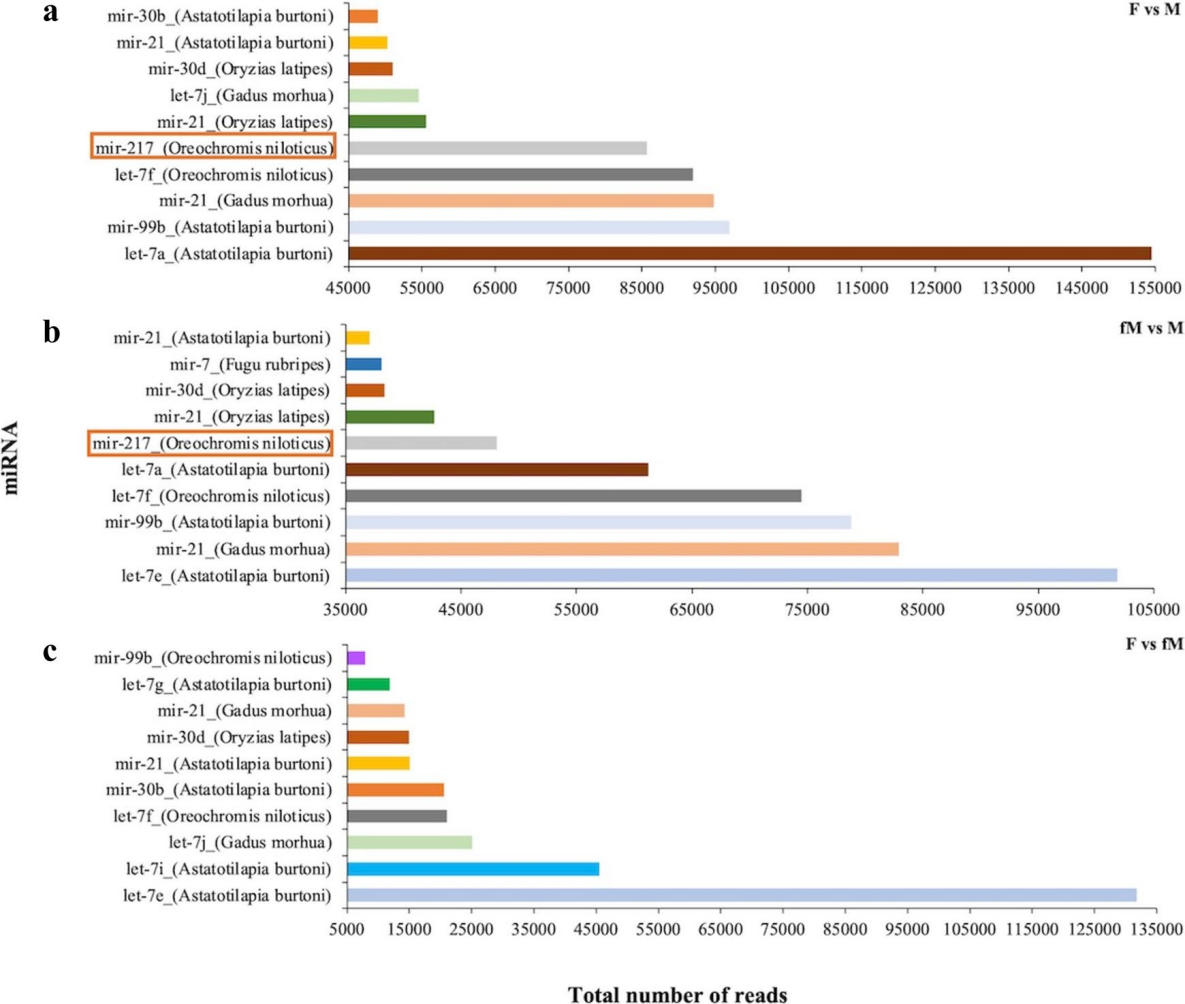
Total number of reads of the ten most abundant differentially expressed miRNAs in the three different gilthead seabream gonad comparisons, between F and M gonads (**a**), between fM and M gonads (**b**) and between F and fM gonads (**c**) during the reproductive season. F represents mature female gonads, M represents the mature and active male parts of male gonads and fM represents the immature and inactive parts of male gonads

Among the three comparisons on the most abundant and significantly differentially expressed miRNAs, only miR-217 was identified to be commonly differentially expressed in two of the comparisons, i.e. F vs M and fM vs M (Fig. 7).

## Discussion

The *fanc* gene family has not yet been the subject of extensive study in teleost fish. Prior to the present study, investigation of the *fanc* gene family in teleost fish had been limited to the zebrafish, with research focusing on the role of these genes during development and in sex determination (Titus et al. 2009; Rodriguez-Mari and Postlethwait 2011). The objective of the present study was to gain further insights into the function of *fanc* genes and their regulation through miRNAs during reproduction. Therefore, we examined gilthead seabream *fanc* gene expressions in (a) the inactive and immature ovarian part of the male gonad, (b) the active and mature testicular part of the male gonad and (c) the exclusively ovarian female gonad. The RNA Bioanalyzer profiles have been found to be indicative of the maturation progression of the obtained samples (Papadaki et al. 2020; Rojo-Bartolome et al. 2016, 2017a, b). Accordingly, in the present study, the 5S/18S marker exhibits higher expression in immature samples compared to mature ones, with an average value of 7.83 in fM gonads containing primary oocytes and 3.74 in F samples undergoing the vitellogenesis stage. To the best of our knowledge, this is the first study on the differential expression of these three genes in gonads in a non-model teleost fish, whereas latest knowledge on the function of the genes relies mainly on their mutations and correlation of the resulting phenotypes with the human FA phenotype. In a recent study in zebrafish, knockouts of 17 *fanc* genes including *fancc*, *fancd2* and *fancl*, led to complete or partial female-to-male sex reversal, without loss of reproductive competence in any of the knockouts (Ramanagoudr-Bhojappa et al. 2018). On the contrary, in mice, both male and female, mutations in the *fancd2* gene have been shown to induce sterility (Nie et al. 2020), stressing the role of *fanc* genes in maintaining the physiological state of the gonad. The findings of the present study indicate a pronounced male-specific expression of *fancl* and *fancd2*, which may point to the existence of a hitherto unidentified male-specific biomarker in teleosts. However, in *Astatotilapia burtoni*, *fancd2* was not found to be differentially expressed between the sexes (Bohne et al. 2016). Within the present work, differential expression was also examined between mature and immature females. Here, a disparity in the expression levels of mature and immature ovarian tissues was observed for *fancl* and *fancc*, with the expression of these genes being higher in the mature ovaries. The third *fanc* gene, *fancd2* exhibited comparable expression levels between the two female gonad stages. In contrast, for two gonochoristic teleost species, the zebrafish and the rainbow trout (*Oncorhynchus mykiss*), studies demonstrated a heightened expression of *fancl* during the initial stages of oogenesis (Baron et al. 2008; Rodriguez-Mari et al. 2010) and absence in the fully mature fish. In light of these findings, further investigations are required to elucidate the differential expression of *fanc* genes with a view to clarifying their role in the reproductive process of teleosts with different reproductive strategies.

Another aim of the present study was to identify miRNAs with a potential regulatory function for the three *fanc* genes. A first comparison of the obtained miRNA profiles between mature and immature ovarian tissues in the present study, revealed similarities between the two stages in female sharpsnout seabream (*Diplodus puntazzo*), another hermaphroditic fish (Papadaki et al. 2020). This confirms that miRNAs do play an important role in reproductive processes. Hierarchical clustering of differentially expressed miRNA, revealed three distinct groups according to their gonadal stages (Fig. 5) comprising well-known gonad-enriched miRNAs like miR-202 and miR-21 (Juanchich et al. 2013; Yan et al. 2021; Bouchareb et al. 2017). In the subsequent downstream analysis, the emphasis was placed on miRNAs that exhibited an inverse expression pattern in comparison to the *fanc* genes. Among them, miR-210, miR-10926 and miR-217 were identified as having also a complete seed region complementarity with *fancd2, fancl* and *fancc*, respectively. It is widely acknowledged that miRNA targeting is contingent upon the base pairing of the seed region to specific sites in mRNA 3'UTR. In addition to the established process of canonical target binding with a perfect seed match of 2–7 nt, evidence has emerged indicating that non-canonical sites may also play a role in regulating mRNA expression. For example, it has been demonstrated that imperfect pairing to the miRNA seed can be compensated for by extended pairing interactions with the 3'-portion of the miRNA. The present study was conducted with the specific objective of identifying appropriate seed pairing in order to detect putative regulatory miRNAs acknowledging that these miRNAs may not be exclusively regulating the three *fanc* genes under investigation. Among the three identified miRNAs, miR-10926 was the sole differentially expressed miRNA to demonstrate upregulation in the immature compared to the mature ovarian stage. Up until today, this miRNA has only been shown to play a role in the oocyte development of the Wanxi white goose *Anser anser* (Li et al. 2024). With regard to miR-210, it is a well-documented and conserved miRNA that plays a role in a number of biological processes, including DNA repair, regulation of the cell cycle and apoptosis (Hui et al. 2019). This miRNA is upregulated in the ovaries of marine medaka (*Oryzias melastigma*) under hypoxic conditions. It has furthermore been shown to target apoptotic genes, thereby suppressing apoptosis (Tse et al. 2015). In a recent study in zebrafish, dissected ovaries were cultured with a miR-210 mimic and among the differentially expressed, downregulated and validated for miR-210 seed region complementarity genes were *fancd2*, *fanci* and *fancl* (van Gelderen and Ribas 2024), in agreement to our study, where miR-210 was found to putatively target *fancd2.*

Regarding miR-217, in the present study it was found to be one of the most abundant differentially expressed miRNAs in gilthead seabream gonads, with higher expression in female gonads, consistent with a study in tilapia (*Oreochromis niloticus*) (Tao et al. 2016), but in contrast to relevant studies in *Acrossocheilus fasciatus* (Wei et al. 2017) and the discus fish (*Symphysodon aequifasciatus*) (Fu et al. 2020), where it was found to be male-biased. The high read counts of this miRNA indicate its possible role in regulating reproduction-related processes in fish. Similarly, high read counts in gonads and a putative role in steroid hormone biosynthesis have also been shown in the discus fish (Fu et al. 2020). Furthermore, in the majority of studies conducted on teleosts, miR-217 has been found to be associated with immune responses (Ahkin Chin Tai and Freeman 2020; Jia et al. 2018; Salifu et al. 2023; Zhang et al. 2020). Since the gilthead seabream is a protandrous species, immune- or apoptosis-related processes may interact with reproductive-related processes to initiate and maintain sex reversal. A similar interaction has been suggested for the protogynous ricefield eel (*Monopterus albus*) (He et al. 2022).

Taken together, the present study demonstrates the dynamic gene expression of three *fanc* genes in the gilthead seabream gonads, pinpointing to their putative role in gonad maturation and sex reversal processes. Furthermore, it reports on the possible regulation of *fanc* genes through miRNAs and it is suggested that three specific miRNAs (namely, miR-210, miR-10926 and miR-217) can potentially target the three *fanc* genes under study. Still, further studies are required to facilitate a comprehensive understanding of the function of *fanc* genes and the mechanisms through which they are regulated.

## Supplementary Information

Below is the link to the electronic supplementary material.

Supplementary file1 (DOCX 15 KB)

Supplementary file2 (XLSX 248 KB)

## Data Availability

No datasets were generated or analysed during the current study.
