# Supplementary material for: Exploring the Fanconi Anemia Gene Expression and Regulation by MicroRNAs in Gilthead Seabream (Sparus aurata) at Different Gonadal Development Stages
Source: Mar Biotechnol (NY). 2025 Apr 11;27(2):74. doi: 10.1007/s10126-025-10444-x (PMC11991948; doi:10.1007/s10126-025-10444-x)
Supplement: Supplementary file 1 — Supplementary file1 (DOCX 15 KB) [file 10126_2025_10444_MOESM1_ESM.docx]

**Supplemental table 1**. Total read counts of mapped and counted reads for each sample, after removal of low-quality reads, adaptor sequences, reads less than 10 nt and total read counts less than 5. F: mature female, M: mature and active male part in male gonad, fM: immature and inactive female part in male gonad.

| Name | Raw data | Trimmed data | Percentage trimmed (%) |
| --- | --- | --- | --- |
| F-1 | 17.790.749 | 13.664.965 | 76,81 |
| F-2 | 8.017.117 | 6.533.452 | 81,49 |
| F-3 | 16.297.684 | 12.810.367 | 78,6 |
| M-1 | 15.780.181 | 12.337.410 | 78,18 |
| M-2 | 15.748.911 | 15.154.347 | 96,22 |
| M-3 | 7.861.559 | 7.335.865 | 93,31 |
| fM-1 | 18.655.576 | 17.185.208 | 92,12 |
| fM-2 | 7.457.032 | 6.433.956 | 86,28 |
| fM-3 | 9.380.542 | 7.797.248 | 83,12 |
